# A graph model to describe the network connectivity of trabecular plates and rods

**DOI:** 10.3389/fbioe.2024.1384280

**Published:** 2024-05-06

**Authors:** Matthias Walle, Diana Yeritsyan, Mohammadreza Abbasian, Ramin Oftadeh, Ralph Müller, Ara Nazarian

**Affiliations:** ^1^ Musculoskeletal Translational Innovation Initiative, Carl J. Shapiro Department of Orthopaedic Surgery, Beth Israel Deaconess Medical Center and Harvard Medical School, Boston, MA, United States; ^2^ Institute for Biomechanics, ETH Zürich, Zürich, Switzerland; ^3^ Department of Orthopaedic Surgery, Yerevan State Medical University, Yerevan, Armenia

**Keywords:** microarchitectural properties, trabecular plate, trabecular rod, graph model, volumetric spatial decomposition, finite element analysis

## Abstract

**Introduction:** The trabecular network is perceived as a collection of interconnected plate- (P) and rod-like (R) elements. Previous research has highlighted how these elements and their connectivity influence the mechanical properties of bone, yet further work is required to elucidate better the deeply interconnected nature of the trabecular network with distinct element formations conducting forces per their mechanical boundary conditions. Within this network, forces act through elements: a rod or plate with force applied to one end will transmit this force to a component connected to the other end, defining the boundary conditions for the loading of each element. To that end, this study has two aims: First, to investigate the connectivity of individually segmented elements of trabecular bone with respect to their local boundary conditions as defined by the surrounding trabecular network and linking them directly to the bone’s overall mechanical response during loading using a mathematical graph model of the plate and rod (PR) Network. Second, we use this model to quantify side artifacts, a known artifact when testing an excised specimen of trabecular bone, where vertical trabeculae lose their load-bearing capacity due to a loss of connectivity, ultimately resulting in a change of the trabecular network topology.

**Resuts:** Connected elements derived from our model predicted apparent elastic modulus by fitting a linear regression (*R* ^*2*^ *= 0.81*). In comparison, prediction using conventional bone volume fraction results in a lower accuracy (*R* ^*2*^ *= 0.72*), demonstrating the ability of the PR Network to estimate compressive elastic modulus independent of specimen size or loading boundary condition.

**Discussion:** PR Network models are a novel approach to describing connectivity within the trabecular network and incorporating mechanical boundary conditions within the morphological analysis, thus enabling the study of intrinsic material properties of trabecular bone. Ultimately, PR Network models may be an early predictor or provide further insights into osteo-degenerative diseases.

## 1 Introduction

Trabecular bone strength depends not only on bone volume but also on the local structure of the trabecular network (Nazarian et al., 2009). Previous research has characterized the trabecular network as a collection of plate-like and rod-like elements, where, per definition, plates are structural elements with small thicknesses compared to the planar dimension, and rods resemble a cylindrical shape. Micro-computed tomography (μCT) can be used to capture the trabecular bone structure, and plates and rods can be identified using a shape-preserving 3D-thinning algorithm (Saha et al., 1994; 1997) followed by a 3D-topological classification (Saha and Rosenfeld, 2000; Saha et al., 2010). Using this method (Stauber and Müller, 2006a; Liu et al., 2011), previous research has identified that spatial and temporal variations of plates and rods are a vital determinant of bone’s mechanical properties, including yield strength and elastic modulus (Liu et al., 2009; Zhou et al., 2014; Wang et al., 2015). Typically, plate-related measures are positively associated with elastic modulus and yield strength (Zhou et al., 2014), with axially aligned plates expressing higher bone mineral density (Liu et al., 2009; Wang et al., 2015). At the same time, most road-related parameters are inversely associated with mechanical properties (Zhou et al., 2014); thus, they have been hypothesized to initiate mechanical failure (Liu et al., 2009). Previous research has shown that plate and rod models, combined with their connectivity (plate-plate, plate-rod, rod-rod connections), represent bone strength and bulk tissue response to mechanical loading more precisely than bone volume (Liu et al., 2008). However, current plate and rod characterizations lack a complete description of the interplay of all components within the trabecular network. Within the topology of plate-like and rod-like structures, forces act through elements; a rod with force applied to the top will transmit most of this load to components connected to its bottom. This plate and rod (PR) network defines the boundary conditions for the loading and unloading of individual elements and is, therefore, key to the mechanical evaluation of trabecular bone. This is evident in side artifacts (Ün et al., 2006), where the specimen excision-induced disruption of the PR network’s peripheral connectivity affects an excised specimen’s mechanical properties. Specifically, in inhomogeneous, anisotropic bone samples, even a single or missing trabeculae may induce catastrophic failure (Stauber et al., 2014). Given the dependence of side artifacts on PR connectivity (Zhu et al., 1994; Andrews et al., 2001; Onck et al., 2001), it may affect specimen strength and elastic modulus differently due to variations in failure mechanisms.

Therefore, we propose the following two aims: 1) to develop a mathematical graph model that links the trabecular PR network to the bulk mechanical properties of the specimen, and 2) to test the model by evaluating the contribution of side artifacts to bulk specimen mechanical properties as a function of the PR network. We will accomplish the former by modeling the connectivity of individually segmented elements in the specimens, with respect to their local boundary conditions as defined by the surrounding trabecular network, and the latter by linking them directly to the overall mechanical response of the specimens during loading. *We hypothesize that the PR network better describes specimen bulk tissue mechanical properties than bone volume*. We used micro-finite element (μFE) analysis derived from μCT scans of bovine trabecular bone specimens to identify bulk tissue properties, validated by compressive mechanical testing. We derived a spatial network of these elements from CT scans to study interconnections between plate and rod objects and used a mathematical graph model to identify load-bearing (*i.e.*, connected) elements. We utilized an inner core specimen model to introduce controlled changes into the trabecular network while maintaining consistent morphological parameters, subsequently extracting sub-volumes from the original specimen. This enabled us to introduce connectivity changes at the cored specimen interface and directly link changes within the trabecular network to changes within the microscopic stress distribution derived from μFE modeling. Ultimately, the interplay of microscopic spatial complexity and heterogeneity variations within the PR network may provide insights into load transmission mechanisms within the trabecular bone and be an important indicator for many osteopathic diseases.

## 2 Materials and methods

### 2.1 Specimen preparation and imaging

**FIGURE 1:**
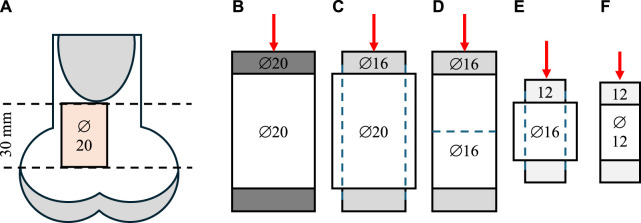
Sample preparation and step-wise reduction approach. **(A)** The location of the specimens excised from bovine femurs. The samples were first tested with 20 mm **(B)** and 16 mm **(C)** plungers at the original full diameter of 16 mm. Then, the samples were cored down to a 16 mm diameter and re-tested with a 16 mm plunger **(D)**. To preserve the aspect ratio, the samples were then cut in half, perpendicular to the long axis, and tested using a 12 mm plunger **(E)**. Finally, the samples were further cored down to 12 mm diameter and tested a third time with a 12 mm plunger **(F)**.

Twelve bovine femurs from 36-month-old adult female animals were obtained from a local butchery. Perpendicular cuts to the longitudinal axis of the femur were performed on frozen samples (Ducheyne et al., 1977; Bentzen et al., 1987) using a band saw and a self-leveling 3-beam line laser (DW089K, DeWalt Industrial Tool Co., Baltimore, MD, United States of America) at the proximal end. Growth plates were avoided using individual visual X-ray assessment. A cylindrical specimen of trabecular bone (∅ = 20 mm) was extracted from each sample using a Magnicon Bond diamond core drill while secured and submerged in water (Keaveny et al., 1993). A low-speed saw (Isomet, Buehler, Lake Bluff, IL, United States of America) with two parallel diamond blades was used to cut uniformly sized (l = 30 mm) specimens (An et al., 1999; Tawy et al., 2016). After coring and cutting, the specimens were subjected to ultrasonic cleaning for 50 min (Tabletop Ultrasonic Cleaners, FS-140, Fisher Scientific, Waltham, MA, United States of America). When not used, samples were wrapped in 0.9% saline-soaked gauze and stored in a freezer at −20°C (Linde and Sørensen, 1993). Specimens were scanned using a μCT (μCT40, Scanco Medical AG, Brüttisellen, Switzerland) at an isotropic voxel size of 30 μm with a tube energy of 55 kVp, a current settings of 72 μA, an integration time of 200 ms, and a 1,200 mg HA/cm^3^ beam hardening correction. Density calibration was performed using a calibration phantom scanned weekly according to the manufacturer’s recommendations (Bouxsein et al., 2010). The sensitivity of network topology to even small changes was investigated by intentionally introducing side artifacts. Specifically, to minimize material-related variability across samples, the same samples were subjected to five non-destructive mechanical testing stages sequentially with three different plunger sizes in a step-wise reduction approach, as outlined in Figure 1. This sequential testing allowed the evaluation of the same samples at three reduced diameters (∅ = 20, 16, 12 mm) and lengths (l = 30, 15 mm) while maintaining a consistent aspect ratio resulting in increased contribution of side-artefacts with decreasing specimen size (Table 1; Lievers et al., 2010).

**TABLE 1:** Sample parameters at different testing stages and the expected contribution of side artifacts.

Test stage	Sample diameter (mm)	Sample length (mm)	Sample size (n)	Plunger diameter (mm)	Side artifact contribution
1	20	30	12	20	Low
2	20	30	12	16	Minimal
3	16	30	12	16	Medium
4	16	15	24	12	Minimal
5	12	15	24	12	High

### 2.2 Network connectivity model

**FIGURE 2:**
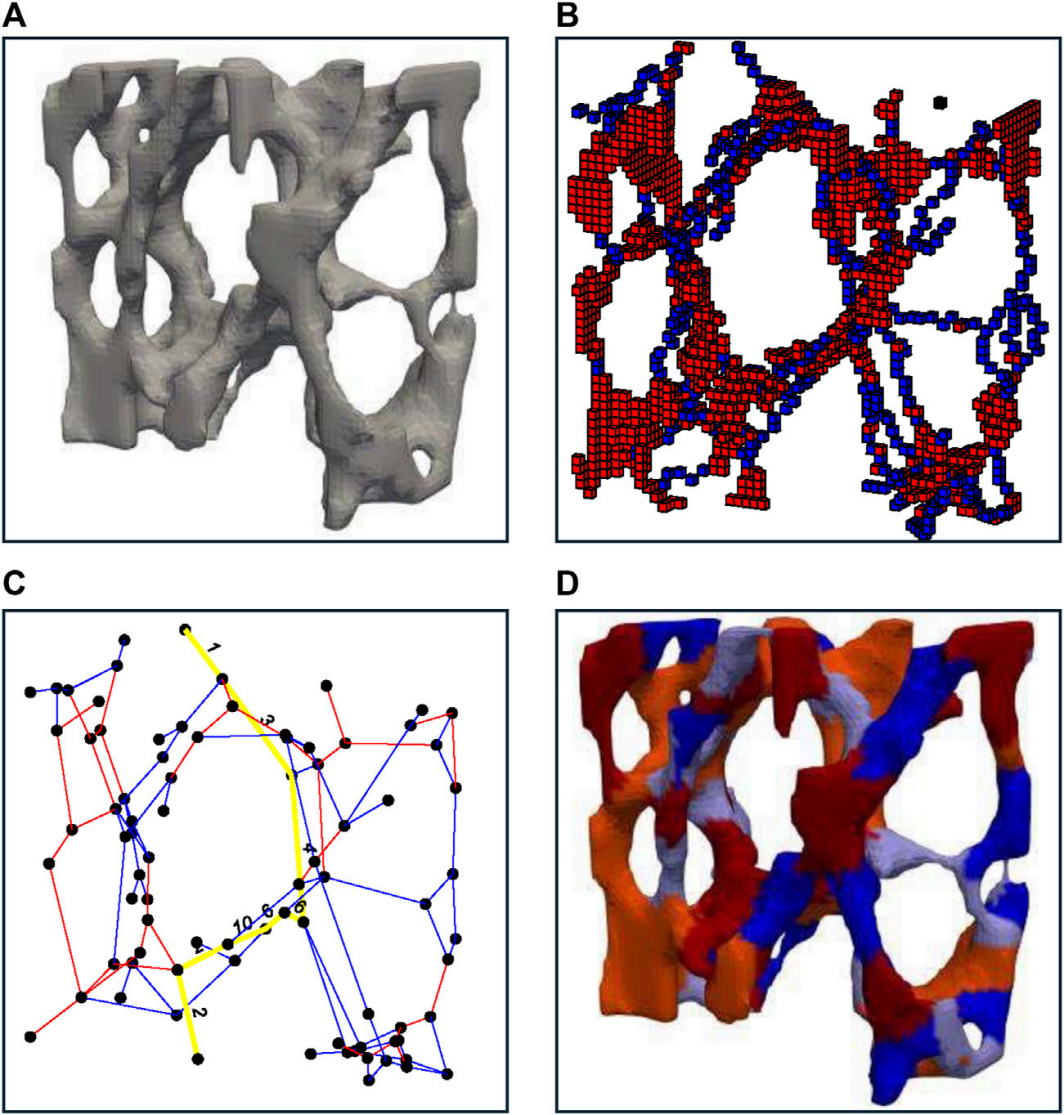
A representative illustration indicating the strategy used for bone decomposition. **(A)** shows an enlarged view of bone morphology. **(B)** shows thinned and classified geometry with identified plate-like structures in shades of red and rod-like structures in shades of blue. **(C)** shows the re-transformed bone geometry with identified plate-like structures in shades of red and rod-like structures in shades of blue with lighter to darker shades denoting individual trabeculae. **(D)** shows the final plate and rod network graph with a possible load transfer path in yellow.

An iterative thinning process, first described by P.K. Saha et al. (Saha and Chaudhuri, 1996), was employed, to produce a shape-preserving representation of the original volumetric image (Figure 2A). The resulting one-voxel-thick skeletons were classified using a three-dimensional digital topological characterization method (Figure 2B; Saha and Rosenfeld, 2000) (additional details provided in a supplemental document). For each voxel, it was determined whether it is a surface voxel, a surface-end voxel, an arc voxel, an arc-end voxel, an arc–arc intersection voxel, an arc–surface intersection voxel, a surface–surface intersection voxel, or an isolated voxel. Following the work by Stauber et al. (Stauber and Müller, 2006a), slender planes were reduced to rods within two iterations, and all rods shorter than four voxels were removed. Within this new optimized image, individual rods and plates were separated by removing all points within a junction point’s radius (r = 2 voxels), enabling individual labeling of rods with odd and plates with even indices. Using a 3D-bitmapping image dilation algorithm described by van den Boomgard et al. (Boomgaard and Balen, 1992), individually labeled images were remapped to their original geometry ( Figure 2C ). Within the original geometry, a voxel was defined as a junction voxel if it belonged to one element and any of its 26 adjacent neighbors belonged to a different element. This resulted in a two-voxel thick junction surface between adjacent elements. Junctions were stored in a sparse, numeric adjacency matrix, where the location of each nonzero entry specified a junction (edges) between two elements (nodes), building the basis of an unweighted network graph ( Figure 2D ). Nodes were defined as junction points between individual trabeculae and edges as individual trabeculae. By applying standard graph analysis algorithms such as the Boykov-Kolmogorov algorithm (Boykov and Kolmogorov, 2001), which computes the maximum flow by constructing two search trees associated with nodes between the source and target, connected elements were identified with respect to a specific loading case. Specifically, trabeculae connected to the proximal and distal surfaces were considered sources and sinks, respectively, creating a multiple sources-sinks maximum flow problem between the boundaries where the compressive force was applied. To simplify this, unbounded-capacity artificial super-nodes s and t were introduced, connecting all nodes on the proximal (collective sources) and distal (collective sink) specimen surfaces, respectively. A weighted bone volume fraction (BV/TV) was derived from the connected bone structure, forming a continuous volume that contributed to the connection between the boundaries. This weight-bearing volume followed the connectivity, layout, and boundary conditions of the trabecular network and the applied load.

### 2.3 Mechanical assessment

To calculate specimen stiffness, μFE analysis was carried out. 3D geometries for linear μFE analysis were generated on a voxel-based approach. Each voxel in the μCT scan was converted to one hexahedral element. The resulting model consisted of approximately 500 million elements. Images were subjected to Gaussian filteration (σ = 1.2) to reduce noise and were binarised at a threshold of 800 mg HA/cm^3^ and assigned Young’s modulus and Poisson’s ratio values of 15 GPa and 0.3 respectively (Tawy et al., 2016). Different-sized metal plungers were virtually attached at the axial ends to apply force. The proximal surface of the bone was deformed by 1% by applying a pure compression force in the axial direction. Each model was solved using ParOSol (Flaig and Arbenz, 2011)running on eight cores on a local machine (i7-6900K LGA 2011, DDR4-3000 PC4-2,400, 16 GB × 8) in under 60 min. Von Mises Stress was calculated at the center of each element (Flaig and Arbenz, 2011). FE models were validated using non-destructive axial compression displacement-driven mechanical tests of up to 0.6% strain using an Instron load frame with plungers of different sizes in unconfined conditions (Linde et al., 1992). A self-adjustable platen with a spherical joint was built to ensure full engagement between the specimen and the plunger for uniform load distribution during compression (Fazzalari et al., 1998; Hernandez et al., 2014). The slope of the linear portion of the pre-yield region was used to estimate the apparent modulus of the trabecular bone specimens (García-Rodríguez et al., 2008). Compressive engineering stress σ and strain ε were calculated using the original plunger, specimen dimensions, and machine head displacement.

### 2.4 Statistical analysis

The Shapiro-Wilk test was used to evaluate data distribution. Receiver-operating characteristics were used to evaluate the relationship between von Mises stress and load-bearing elements identified by the PR Network model. True-positive (TPR) and false-positive (FPR) rates were calculated at all thresholds by comparing predictions by the PR network to predictions of the μFE model, followed by the calculation of the area under the curve (AUC). Pearson moment correlation coefficients were calculated to evaluate the capability of the connected bone volume identified by the PR network when weakly connected trabeculae were present due to side artifacts. The statistical significance of the correlation coefficient was assessed using a Student’s t-test. All statistical tests were performed with Matlab R2020a (The MathWorks, Inc., Natick, MA, United States). Data are presented as the mean (± standard deviation) unless otherwise reported, and *p* < 0.05 was considered significant.

## 3 Results

**FIGURE 3:**
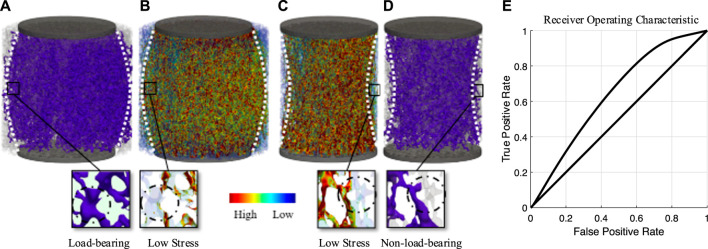
A representative image indicating the identification of load-bearing elements identified by maximum flow between the plungers. **(A)** shows load-bearing elements (purple) for a plunger smaller than the specimen. **(B)** shows μFE analysis of the same loading case, showing additional load-bearing elements in the PR Network that highlight the relevance of lower stress trabeculae. **(C)** shows μFE analysis for a plunger with the same size as the specimen. **(D)** shows load-bearing elements (purple) for this loading case, highlighting non-load-bearing elements at the specimen interface. **(E)** ROC curve showing the true-positive rate of identifying a load-bearing element using the PR Network against the false-positive rate at various Mises stress threshold settings derived from µFE.

For the three sample diameter-based groups, two different load cases were distinguished: first, compressive loading with a plunger of the same size as the specimen, and second, compressive loading with a plunger with a smaller diameter than that of the sample. Figure 3 illustrates the load transfer of both cases in (A) and (B), as well as (C) and (D), respectively, by using the PR Network in (A) and (D) and comparing it directly to voxel-based μFE models in (B) and (C). As seen for case (A), the trabecular network distributed the local load, exceeding the volume under the plunger, resulting in a convex loading pattern to reduce stress on individual elements. However, this distribution was not always uniform and highly depended on the layout of the trabecular network. Case (D) shows the same specimen after re-coring. This direct change within the system led to a loss in connectivity for edges located at the interface region, resulting in an hourglass shape. The area under the curve analysis derived from the ROC analysis suggested acceptable discrimination (AUC = 0.73) between the PR Network and mechanical signal (Figure 3E). This indicates that the PR Network and μFE models, while correlated, identify some distinct sets of load-bearing elements. Specifically, the PR Network identifies a continuous pathway of interconnected elements that transfer loads between the platens. In contrast, the μFE model identifies high stress elements that may not necessarily form an interconnected load-transferring network (Figure 3).

**FIGURE 4:**
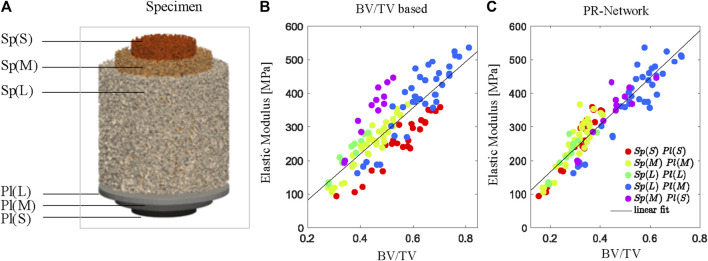
The PR Network estimates stiffness independent of specimen size based on the weighted bone volume fraction (BV/TV) identified by the model. **(A)** Combinations of specimen size Sp for small S, medium M, and large L samples and plunger size (Pl) for small (S), medium (M), and large (L) plungers used for axial BV/TV determined using conventional measurements **(B)** and the PR Network **(C)**. Color codes indicate grouped combinations of specimen and plunger size.

For all different specimen geometries (*Sp*) and plunger combinations (*Pl*), the PR Network predicted apparent moduli by fitting a linear regression (*R* ^*2*^ *= 0.81*, *p* < 0.05). In comparison, prediction using conventional bone volume fraction results in a lower accuracy (*R* ^*2*^ *= 0.72*, *p* < 0.05), demonstrating the ability of the model to estimate compressive elastic modulus independent of specimen size or loading boundary condition (Figure 4). The μFE derived apparent Young’s modulus (E_FE_) was linearly related (R^2^ = 0.85, *p* < 0.05) to the experimentally measured apparent Young’s modulus (E_meas_).

## 4 Discussion

We introduced the Plate and Rod (PR) Network, a novel network characterization to describe connectivity within the trabecular network of individually segmented plate- and rod-like elements and their inter-connected character. The PR Network approach provided a functionally representative bone volume evidenced by a more accurate assessment of compressive elastic modulus of excised specimen than traditional bone volume fraction methods. This is due to its ability to account for the interconnectivity of high-potential load-bearing elements along the main loading axis.

In contrast to previous approaches, the PR Network evaluates connectivity with respect to global and local boundary conditions. For instance, a previous study employed multi-regression models and local morphological plate and rod measures, including their connections (plate-plate, plate-rod, rod-rod) to predict the mechanical properties of trabecular bone (Liu et al., 2008). Other commonly used measures in micro-CT include connectivity density (Bouxsein et al., 2010). Unlike these previous methods that focused on local measures like trabecular morphology or connectivity density and improved predictions over just using BV/TV, our goal with the PR Network was to emphasize the significance of higher-order connections between these trabecular bone elements in determining mechanical competence. This interrelation has just become a subject of more recent studies and has shown to be useful for identifying biomarkers for clinical procedures (Mondal et al., 2019; Nguyen et al., 2019). By evaluating the connectivity with respect to global and local boundary conditions, the developed PR Network increases the influence of channels of high-potential elements, stretching from source to sink. This includes channels of high-volume elements ranging from proximal to the distal end along the vertical axis of the specimen. Therefore, the PR Network has the potential to provide a novel approach to characterize mechanical behavior and could aid in the assessment of bone strength.

The sensitivity of network topology to even small changes was demonstrated by intentionally introducing side artifacts. Alteration or damage to bone tissue at the edges of an excised trabecular specimen can profoundly impact its mechanical stability (Ün et al., 2006). However, this well-known “side artifact” also presents an opportunity to evaluate the accuracy of our computational modeling approach. To intentionally introduce side artifacts, we sequentially reduced specimen dimensions and increased the proportional damage at the interface. This enabled the assessment of the model’s ability to predict resultant changes in trabecular connectivity and mechanics. Although bone volume fraction is considered the primary predictor of elastic modulus in porous media like trabecular bone (Nazarian et al., 2008a; Oftadeh et al., 2015), subtle network alterations may significantly influence bone strength. Indeed, our results showed that small connectivity changes in bovine trabecular networks significantly affected mechanical competence. Nevertheless, the PR Network model still accurately estimated compressive elastic modulus independent of size or boundary conditions. These insights highlight the complex interdependence of trabecular connectivity and mechanics. The model demonstrated robust utility despite intentionally introduced artifacts, showcasing its potential for the development of more advanced computational models for trabecular bone.

The developed PR Network approach could be highly valuable for studying bone diseases like osteoporosis, in which deteriorating trabecular connectivity increases fracture risk. Specifically, this approach could identify which structural elements are most crucial for load transfer within the trabecular bone network. Mapping the connectivity in this way may enable the assessment of how progression and loss of key trabecular members elevate fracture risk. While bovine bone was used here, the model may be translatable to human applications using high-resolution CT imaging modalities like high-resolution peripheral quantitative computed tomography (HR-pQCT) (Manske et al., 2015). Bovine and human bone share similar structural properties like porosity, trabecular/cortical content, and mechanics (Fletcher et al., 2018). Further, plate and rod segmentation has been applied using HR-pQCT imaging of human bone at 61 μm resolution [37]. Combined with clinical imaging methods, the PR Network could enable the assessment of fracture risk and treatment efficacy in osteoporosis. However, the plate classification process may require further optimization as an additional step was introduced to convert narrow plates to rods (Stauber and Müller, 2006a). Future efforts should evaluate the sensitivity of the decomposition method. This study demonstrates the initial capabilities of this method, and with optimization for human application, the PR Network approach may provide novel views on osteoporosis mechanisms and guiding patient care.

Some limitations should be noted. The mechanical properties of bone change significantly depending on whether the specimens are tested *in situ* or *ex situ* (Carter and Hayes, 1977; Linde and Sørensen, 1993). However, replicating the neighboring tissue’s degree of restriction *in vivo* is challenging. Unrestricted test conditions were used to improve comparability with other studies. Further, local strain measurement has yielded better accuracy for validating the FE model. However, the FE model used in our study has been widely applied in previous bone research (Van Rietbergen et al., 1998; Bouxsein et al., 2010). Combining empirical evidence from mechanical testing with this extensively validated FE model provides adequate fidelity for the present investigation. To prevent buckling, all specimens were kept within the recommended height, cross-sectional area, and aspect ratio range of 1–2 (Wang et al., 2010). Consequently, no regional strain measurement was required. Regarding computational limitations, the FE model only considered the structure and did not include variations in the local material properties of the bone matrix. Also, we assumed a simplified linear elastic FE model and materials to derive local stress, which is only valid for small deformations. In addition, the geometry of the FE model may be sensitive to image segmentation using automatic thresholds, leading to thin structures, which may behave too stiff when bent. Further, it is essential to note that we only compared structural properties with the stiffness determined by the elastic modulus in this study. However, other mechanical properties, such as toughness, z-strength, or torsional bone failure, may be equally important (Nazarian et al., 2008b). Our motivation to investigate elastic modulus was based on findings from other studies (Fyhrie and Schaffler, 1994; Keller, 1994), which show that bone modulus correlates relatively well with bone strength and hardness. Finally, we presented correlations between BV/TV and elastic moduli by pooling data from all specimen groups. While our aim was to underscore the broad applicability of the proposed method, it is important to acknowledge that the analysis involved non-independent samples. The presence of potential autocorrelation effects should be considered, given the lack of complete independence among the samples.

In conclusion, PR Network models are a novel approach to describing connectivity within the trabecular network, incorporating load transfer mechanisms and mechanical boundary conditions within the morphological analysis, thus enabling the study of intrinsic material properties of trabecular bone. PR Network models may be an early predictor or provide further insights into osteo-degenerative diseases.

## Data Availability

The raw data supporting the conclusion of this article will be made available by the authors, without undue reservation.
