# Supplementary material for: A graph model to describe the network connectivity of trabecular plates and rods
Source: Front Bioeng Biotechnol. 2024 May 6;12:1384280. doi: 10.3389/fbioe.2024.1384280 (PMC11103010; doi:10.3389/fbioe.2024.1384280)
Supplement: Supplementary file 1 [file DataSheet1.pdf]

A. Supplement: A Graph Model to Describe the Network Connectivity of Trabecular Plates and Rods

A.1. 3D Digital Topology Method

A.1.1. General Definitions

A volumetric digital image is represented by integer values in a scalar-valued m-by-n-by-p image which may be expressed in Cartesian coordinates (x, y, z). The following definitions in accordance to P.K. Saha et. al [1, 2, 3, 4, 5] are used within this work:

3D-Digital Topology will be carried out in the 26-neighborhood of each voxel p of the 3D image. All 26-adjacent points of a point p will be called 26-neighbors and define the $3 * 3 * 3$ neighborhood N_p of p. The set of points including all 26-neighbors of p excluding p will be denoted as N_p^* as seen in Figure A.1.

The following points are distinct points within this neighborhood based on their adjacency to p. Within the 26-neighborhood there are 6 s-points, 12 e-points, and 8 v-points, making up 26 voxels in total.

1. p_2 is an **v-point** to p_1 if it is 26-adjacent where
   $max\{|x_1 - x_2|, |y_1 - y_2|, |z_1 - z_2|\} \leq 1$
2. p_2 is an **e-point** to p_1 if it is 18-adjacent where
   $max\{|x_1 - x_2|, |y_1 - y_2|, |z_1 - z_2|\} \leq 1$ and $|x_1 - x_2| + |y_1 - y_2| + |z_1 - z_2| \leq 2$
3. p_2 is an **s-point** to p_1 if it is 6-adjacent where
   $|x_1 - x_2| + |y_1 - y_2| + |z_1 - z_2| \leq 1$

We will call two s-points opposite if they are 6-adjacent but not 26-adjacent. Otherwise, they are called non-opposite.

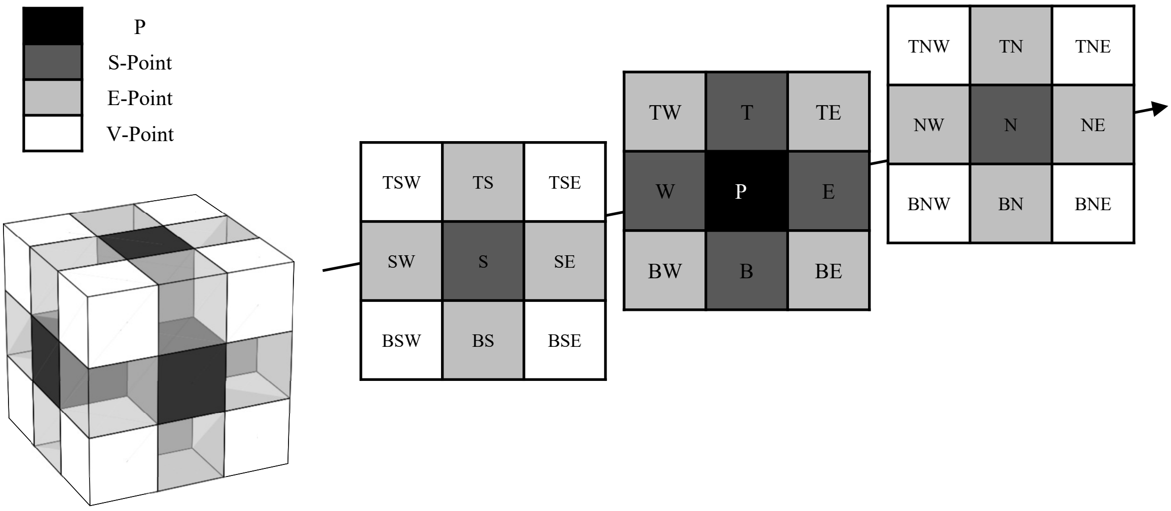

Figure A.1.: Illustration indicating the 26-neighbourhood of an exemplary point p. Each individual point is labeled according to their relative location top using cardinal points (N: North, E: East, S: South, W: West, T: Top, B: Bottom). Gray-scale code indicating 6 s-points, 12 e-points, and 8 v-points making up the 26-neighborhood.

We will assume 26-connectivity between objects considered bone, called black points, and 6-connectivity between objects considered soft-tissue objects, called white points.

The following functions define distinct e-points and v-points based on their location which may be described uniquely by their three non-opposite neighboring s-points a, b, c of N_p.

- $e(a, b, p) = q | q \in N_p^*$ and 6-adjacent to both s-points a, b
- $v(a, b, c, p) = q | q \in N_p^*$ and 6-adjacent to $e(a, b, p)$, $e(b, c, p)$, and $e(c, a, p)$

The following functions are used to expand the $3 * 3 * 3$ neighborhood and can be defined in a $5 * 5 * 5$ neighborhood of p. They are mainly used to preserve sharpness of corners during identification of surface voxels (Figure A.2).

- $f_1(a, p) = q | q \notin N_p$ and 6-adjacent to a.
- $f_2(a, b, p) = q | q \notin N_p$ and 6-adjacent to $f_1(a, p)$ and $e(a, b, p)$.
- $f_3(a, b, p) = q | q \notin N_p$ and 6-adjacent to $f_2(a, b, p)$ and $f_2(b, a, p)$.

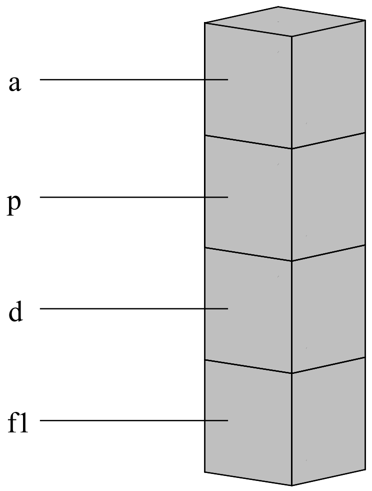

Figure A.2.: Example showing two opposite s-points a and d of point p. function $f1(a,p)$ can be used to extend the 26-neighborhood of p

Above definitions for distinct points will be used frequently by the algorithm and do not change within N_p and its expansion defined by f_1, f_2, f_3. It is most efficient to compute them once and explicitly define them as a function of each distinct s-point combination. Once computed values can be saved in look-up tables and made easily accessible.

Thick-points: thick-points are part of a 2-voxel thick surface which can be identified using the definitions f_1,f_2 and f_3. Thick-points cannot be eroded during primary thinning, as peeling of a 2-voxel thick surface would result in a hole.

Surface points

The later described algorithm defines a peeling process where the surface of the 3D-Image is removed layer by layer. The surface of the image can be defined as the sum of the following s-open, e-open and v-open points:

- A bone voxel is considered an s-open point if at least one s-point of N_p is white before the iteration.
- A black voxel is considered an e-open point if it is not a s-open point and an e-point $e(a,b,p)$ is white while $f_1(a,p)$, $f_1(b,p)$ are black before the iteration.

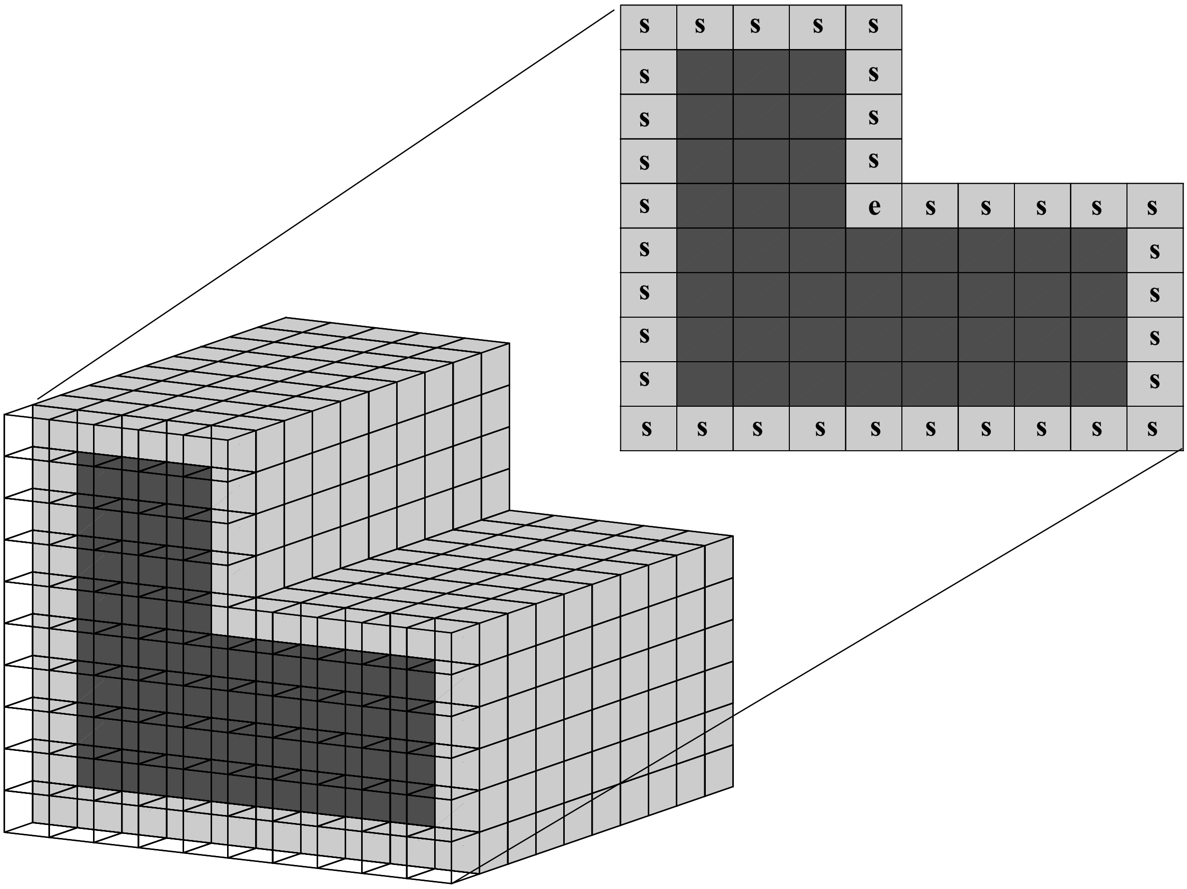

Figure A.3.: Illustration indicating surface identification process. The surface is defined as the sum of all s-open (s), e-open (e) and v-open (v) points.

- A black voxel is considered a v-open point if it is neither a s-open nor e-open point and a $v(a, b, c, p)$ is bone while $f_1(a, p)$, $f_1(b, p)$, $f_1(c, p)$ are black before the iteration.

It is understood from the above definitions that the labeling of points as s-open, e-open and v-open points is made once before each iteration.

Shape preserving points

During primary thinning layers are removed until only shape preserving points remain and the 3D-image is reduced to an one- or two- voxel thick surface. In order

to identify whether a point is a shape preserving point in its neighborhood the following definitions may be used.

Within the $3 * 3 * 3$ neighborhood a middle plane between two opposite s-points a and d can be defined in such way that it does not include a nor d. We will define the following conditions for all three pairs of opposite s-points (a,d), (b,e) and (c,f). Let condition.

- C_1 be $x \in \{b,e,c,f\}$,
- C_2 be $x \in \{b,e,c,f\}$ and x,y are non-opposite,
- C_3 be $x \in \{b,e,c,f\}$ and $x \in \{p_B, p_S, p_W\}$,
- C_4 be $x \in \{b,e,c,f\}$, x,y are non-opposite and $x \in \{p_B, p_S, p_W\}$,
- C_5 be $x \in \{b,e,c,f\}$, x,y are non-opposite and $x,y \in \{p_B, p_S, p_W\}$.

A middle plane $M(a,d,p)$ of N_p in the $3 * 3 * 3$ neighborhood and an extension of this plane $EM(a,d,p)$ in the $5 * 5 * 5$ neighborhood can be defined in the following way:

$M(a,d,p) = \{x|C_1\} \cup \{e(x,y,p)|C_2\}$

$EM(a,d,p) = M(a,d,p) \cup \{f_1(x,p)|C_3\} \cup \{f_2(x,y,p)|C_4\} \cup \{f_3(x,y,p)|C_5\}$

We also define a surface of N_p as follows:

$surface(a,p) = \{a\} \cup \{e(a,x,p)|C_1\} \cup \{v(a,x,y,p)|C_2\}$.

Shape preserving points fulfil the following conditions:

- in N_p two opposite s-points (a,d) exist such that $EM(a,d,p)$ contains a 6-closed path of white points encircling p and each of $surface(a,p)$ and $surface(d,p)$ contains at least one black point before the iteration.
- in N_p a pair of opposite s-points (a,d) exists such that $d \in \{p_e, p_s, p_w\}$, a is white, d or $f_1(d,p)$ is white and each of the sets

$$\begin{gathered}\{e(a,b,p),b,e(b,d,p)\},\{e(a,c,p),c,e(c,d,p)\},\{e(a,e,p),e,e(d,e,p)\},\\ \{e(a,f,p),f,e(d,f,p)\},\{v(a,b,c,p),e(b,c,p),v(b,c,d,p)\},\\ \{v(a,b,f,p),e(b,f,p),v(b,d,f,p)\},\{v(a,c,e,p),e(c,e,p),v(c,d,e,p)\},\\ \{v(a,e,f,p),e(e,f,p),v(d,e,f,p)\} \end{gathered} \tag{A.1}$$

contains at least one black point before the iteration.

Simple points

Erodible points are called simple points. The following definitions can be used first to identify simple points, second to classify the image later on.

Black objects $\varepsilon(p)$, tunnels $\eta(p)$ and cavities $\delta(p)$: We will use definitions of local topological parameters: the number of black objects $\varepsilon(p)$, the number of tunnels $\eta(p)$ and the number of cavities $\delta(p)$ within N_p in order to classify distinct points of our 3D image (inner-surface points, outer-surface points, inner-arc points, arc-end points, surface-surface connection, surface-arc connection, arc-arc connection and isolated points).

- $\varepsilon(p)$ is equal to the number of 26-connected black points within N_p^*
- $\eta(p)$ is one less than the number of 6-connected white components which intersect with an s-point of N_p.
- $\delta(p)$ is always equal to 1 except when all s-points are black points.

The following four conditions were used to define a **simple point**:

- K_1 be N_p^* contains no cavity ($\delta(p) = 0$)
- K_2 be the number of tunnels in N_p^* is zeros ($\eta(p)$)
- K_3 be N_p^* contains one 26-connected black component
- K_4 be p has at least one black 26-neighbor

Classification of a 1-voxel thick skeleton

The following definitions will be used in order to classify the resulting 1-voxel thick skeleton and identify distinct geometric points. Commonly, the following types are distinguished: isolated points (I), inner-arc points (IA), edge-arc points (EA), arc-arc junctions (AA), inner-surface points (IS), edge-surface points (ES), surface-surface junctions (SS) and surface-arc junctions (SA).

Geometric classes: As shown by Saha et al., these topological parameters (A.1.1) are independent of a surface within N_p when the s-point within that surface is black. The remaining points will be called effective points. Each configuration of s-points results in a unique effective point configuration c_{eff}. The resulting configurations can be divided into 10 possible geometric classes depending on their s-point configuration. Within each class, the geometric parameters are identical. These classes are mainly

used to help reducing computational cost. However, they do not directly respond to previously mentioned geometric points yet.

- Class 0: All s-points are bone. Number of effective voxels $n_e = 0$.
- Class 1: Five s-points are bone. Number of effective voxels $n_e = 0$.
- Class 2: Two pairs of opposite s-points are bone. Number of effective voxels $n_e = 0$.
- Class 3: One pair of opposite and two non-opposite s-points are bone. Number of effective voxels $n_e = 1$.
- Class 4: One pair of opposite and another s-point are bone. Number of effective voxels $n_e = 2$.
- Class 5: Three non-opposite s-points are bone. Number of effective voxels $n_e = 4$.
- Class 6: One pair of opposite s-points are bone. Number of effective voxels $n_e = 4$.
- Class 7: Two non-opposite s-points are bone. Number of effective voxels $n_e = 7$.
- Class 8: Only one s-point is bone. Number of effective voxels $n_e = 12$.
- Class 9: No s-point is bone. Number of effective voxels $n_e = 20$.

The number of possible effective point configurations is equal to 2^{n_e}. The following parameters can be immediately identified for Class 0: $\varepsilon(p)$ $\eta(p)$ $\delta(p)$, Class 1: $\varepsilon(p)$ $\eta(p)$ $\delta(p)$, Class 2: $\varepsilon(p)$ $\eta(p)$ $\delta(p)$. For all other classes parameters can be computed once and saved within a lookup table for each class in order to speed up the code.

Classification: Classification can be divided into two steps: initial classification based on $\varepsilon(p)$, $\eta(p)$, $\delta(p)$ according to A.1 and final classification based on the 26-neighborhood of our initial classification accordingly to A.2.

Subfields

The 3D-thinning algorithm describing a peeling process, where each iteration the outer layer is removed is inherently serial. However, it is possible to parallelize the point classification of erodible or shape preserving points, during each iteration. These points are evaluated based on their 26-neighborhood. Parallel removal of two

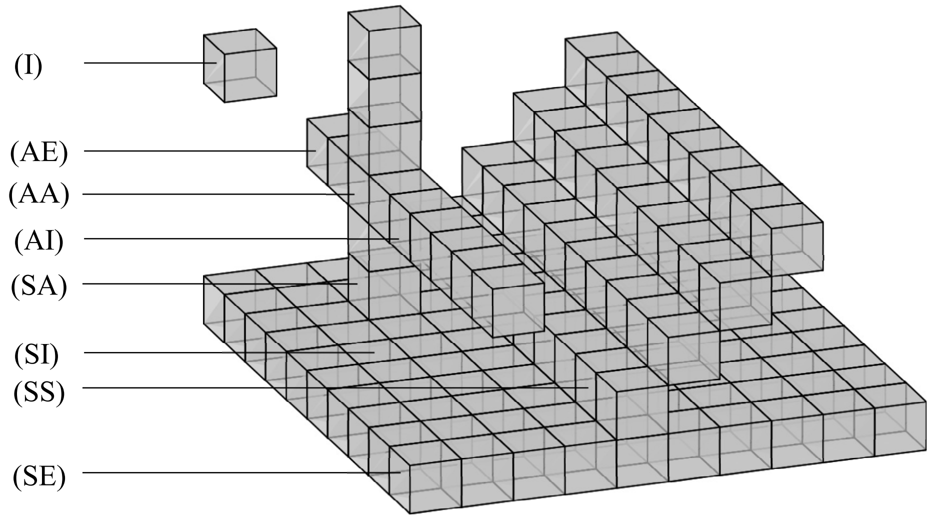

Figure A.4.: The following distinct topological types are distinguished: isolated points (I), inner-arc points (AI), edge-arc points (AE), arc-arc junctions (AA), inner-surface points (SI), edge-surface points (SE), surface-surface junctions (SS) and surface-arc junctions (SA).

points within the same 26 neighborhood could lead to a loss of shape preserving points resulting in a deprived skeleton. The concept of subfields was introduced by Golay et al. [6] and Saha et al. [3] and can be used to partially parallelize the algorithm. The 3D-image is divided in 26 unconnected regions, where within each region none of the points are contained in each other's 26-neighborhood. Each region can be processed in parallel. However, the currently processed image needs to be updated after processing each group.

A.1.2. The Thinning Algorithm

Three-dimensional images of successive micro-CT scans were reconstructed at an isotropic resolution of 30 m, Gauss-filtered (= 1.2), and binarized using a threshold at 600 mgHA/cm3. The following iterative process was first described by PK. Saha et al. [2] producing a shape preserving representation of the original volumetric image. Within this thinning approach, two steps can be differentiated: primary and final thinning. All required definitions required for an implementation of this thinning algorithm can be found in A.1.1.

Primary thinning is a process where the complete image is scanned several times

$\varepsilon(p)$	$\eta(p)$	$\delta(p)$	Initial Class	Poss. Outcome
0	0	0	I_1	$\{I\}$
1	0	0	I_2	$\{ES, EA\}$
2	0	0	I_3	$\{A\}$
> 2	0	0	I_4	$\{AA\}$
1	1	0	I_5	$\{IS, AA\}$
> 1	≥ 1	0	I_6	$\{IS, SA, AA\}$
1	> 1	0	I_7	$\{IS, SA, AA\}$
1	0	1	I_8	$\{IS, SA, AA\}$

Table A.1.: Initial Classification based on $\varepsilon(p)$, $\eta(p)$, $\delta(p)$. Possible outcome: isolated points (I), inner-arc points (IA), edge-arc points (EA), arc-arc junctions (AA), inner-surface points (IS), edge-surface points (ES), surface-surface junctions (SS) and surface-arc junctions (SA)

processing the entire traversal of the image. During each scan, the image border is identified as the aggregate of all s-open, e-open and v-open points. The above-mentioned voxels are either eroded when they are considered simple points or preserved when they are considered a shape preserving point. At the beginning of the algorithm the value "0" is assigned to bone voxels and the largest negative 32-bit integer ($-intmax$) is assigned to marrow voxels. Zero-value voxels denote unmarked bone voxels, which are considered for erosion during each scan of the image. If an unmarked bone voxels is considered shape preserving it is marked, assigned the current iteration number i and cannot be deleted during primary thinning. Otherwise, if it is considered a simple point it is deleted by assigning the value $thr = -maxint + i$. As a result, one single physical image can be interpreted at different stages of the erosion process. Where simple points are always derived from the current image version $I \geq thr$, shape preserving points are derived from the image at the beginning of the current iteration $I > thr$. The erosion process continues until two consecutive iterations contain the same voxel configuration, meaning no more voxel can be eroded or preserved. Primary thinning may results in a 2-voxel thick skeleton, which can be properly reduced using the following final thinning procedure.

Final thinning is a single iteration procedure reducing all thick points of a two voxel thick surface. During this final scan, all marked and unmarked bone voxel are considered for erosion. Thick points cannot be removed during primary thinning, as peeling of a two-voxel thick surface would remove the entire surface and may result in a false representation of the skeleton.

Initial class	26-neighborhood	Final point type
N_2	$\sum BV = 1$	EA
N_2	$\sum BV > 1$	ES
N_5	$N(BV) \in \{N_3, N_4\}$ true for all BV	AA
N_5	$N(BV) \in \{N_3, N_4\}$ true for some BV	IS
N_6	$N(BV) \in \{N_3, N_4\}$ true for all BV	AA
N_6	$N(BV) \in \{N_3, N_4\}$ true for some BV	AS
N_6	$N(BV) \in \{N_3, N_4\}$ true for none BV	SS
N_7	$N(BV) \in \{N_3, N_4\}$ true for all BV	AA
N_7	$N(BV) \in \{N_3, N_4\}$ true for some BV	AS
N_7	$N(BV) \in \{N_3, N_4\}$ true for none BV	SS
N_8	$N(BV) \in \{N_3, N_4\}$ true for all BV	AA
N_8	$N(BV) \in \{N_3, N_4\}$ true for some BV	AS
N_8	$N(BV) \in \{N_3, N_4\}$ true for none BV	SS

Table A.2.: Final Classification. Analysis based on the 26-neighborhood of the initial classification of all bone voxels (BV) in table A.1. Final outcome: isolated points (I), inner-arc points (IA), edge-arc points (EA), arc-arc junctions (AA), inner-surface points (IS), edge-surface points (ES), surface-surface junctions (SS) and surface-arc junctions (SA)

A.1.3. Classification

The resulting one voxel thick skeletons were classified using a three-dimensional digital topological characterization method [4]. For each voxel, it is determined whether it is a surface voxel, a surface end voxel, an arc voxel, an arc-end voxel, an arc–arc intersection voxel, an arc–surface intersection voxel, a surface–surface intersection voxel, or an isolated voxel. A description of the basic principles of this algorithm can be found in A.1.1.

In accordance with Stauber et al. [7], slender planes were reduced to rods within two iterations and all rods shorter than four voxels were removed. Within this new optimized image individual rods and plates were separated by removing all points within a radius (r=2) of a junction point enabling individual labeling of rods with odd and plates with even indices. Using a 3D-bitmapping image dilation algorithm described by van den Boomgard et al. individually labeled images were remapped to their original geometry.

Within the original geometry, a point was identified as a junction point when at least one 26-adjacent voxel of one element was contained within a neighboring plate-

or rod-element resulting in a two-voxel thick junction surface between neighboring elements. Junctions were stored in a sparse, numeric adjacency matrix, where the location of each nonzero entry specifies a junction (edges) between two elements (nodes) building the basis of an unweighted network graph.

A.2. Spatial Network

A graph is a collection of nodes and edges with the following relationships: Nodes are vertices that correspond to objects and edges are connections between objects. Graph edges may have weights and may indicate the potential or strength of each connection. As these models are very general, the specific meaning of nodes, edges and weights highly depends on their application. Further, two types of graphs can be distinguished: undirected and directed graphs. In a directed graph, edges can only be traversed in one defined direction. Undirected graphs have edges that do not have a direction. The edges indicate a two-way relationship, in that each edge can be traversed in both directions.

For the spatial plate and rod network, nodes were defined as junction points between individual trabeculae and edges as trabeculae themselves. Therefore, weights can be chosen according to individual distinct properties (such as size, width, length, density) of each trabeculae. By applying common graph analysis algorithms such as the Boykov-Kolmogorov algorithm, which computes the maximum flow by constructing two search trees associated with nodes s and t, geometric parameters can be evaluated in regards to a specific loading case. In order to evaluate the flow through the whole specimen nodes s and t are artificial super-nodes connected to all nodes on the proximal and distal specimen surface respectively.

Bibliography

[1] P. Saha, B. Chaudhuri, B. Chanda, and D. D. Majumder. "Topology preservation in 3D digital space". In: *Pattern Recognition* 27.2 (1994), pp. 295–300. ISSN: 0031-3203. DOI: 10.1016/0031-3203(94)90060-4.

[2] P. Saha and B. Chaudhuri. "3D Digital Topology under Binary Transformation with Applications". In: *Computer Vision and Image Understanding* 63.3 (1996), pp. 418–429. ISSN: 1077-3142. DOI: 10.1006/cviu.1996.0032.

[3] P. Saha, B. Chaudhuri, and D. D. Majumder. "A new shape preserving parallel thinning algorithm for 3D digital images". In: *Pattern Recognition* 30.12 (1997), pp. 1939–1955. ISSN: 0031-3203. DOI: 10.1016/s0031-3203(97)00016-2.

[4] P. K. Saha and A. Rosenfeld. "Determining simplicity and computing topological change in strongly normal partial tilings of R2 or R3". In: *Pattern Recognition* 33.1 (2000), pp. 105–118. ISSN: 0031-3203. DOI: 10.1016/s0031-3203(99)00037-0.

[5] P. K. Saha, Y. Xu, H. Duan, A. Heiner, and G. Liang. "Volumetric Topological Analysis: A Novel Approach for Trabecular Bone Classification on the Continuum Between Plates and Rods". In: *IEEE Transactions on Medical Imaging* 29.11 (2010), pp. 1821–1838. ISSN: 0278-0062. DOI: 10.1109/tmi.2010.2050779.

[6] M. Golay. "Hexagonal Parallel Pattern Transformations". In: *IEEE Transactions on Computers* C-18.8 (1969), pp. 733–740. ISSN: 0018-9340. DOI: 10.1109/t-c.1969.222756.

[7] M. Stauber and R. Müller. "Volumetric spatial decomposition of trabecular bone into rods and plates—A new method for local bone morphometry". In: *Bone* 38.4 (2006), pp. 475–484. ISSN: 8756-3282. DOI: 10.1016/j.bone.2005.09.019.
